# Supplementary material for: Are carbon emissions trading and green financial instruments synergistic? -Comprehensive quantitative research based on content analysis
Source: PLoS One. 2024 Mar 7;19(3):e0298601. doi: 10.1371/journal.pone.0298601 (PMC10919601; doi:10.1371/journal.pone.0298601)
Supplement: S1 Data — (ZIP) [file pone.0298601.s001.zip › Data/地级市Z_F工作报告.zip!/╜¡╦╒/╦▐╟¿╩╨/└┤╫╘╣1⁄2╓┌║┼í╛┬φ┐╦╩2╛▌═°í┐_2014.docx]

各位代表：

现在，我代表宿迁市人民政府，向大会作工作报告，请予审议，并请各位政协委员和其他列席人员提出意见。

2013年工作回顾

过去的一年，在省委、省政府和市委的坚强领导下，在市人大、市政协的监督支持下，市政府深入贯彻落实党的十八大及十八届三中全会精神，紧紧围绕建设小康社会的奋斗目标，按照“稳中求进、好中求快”的总体要求，大力开展统筹“三大发展”、推进“九比竞赛”活动，经济社会实现了平稳健康发展。主要指标再上新台阶，预计实现地区生产总值1705亿元、增长12.5%，财政总收入381.9亿元、增长14.6%，公共财政收入185.1亿元、增长17.1%，固定资产投资1290亿元、增长25.8%，贷款余额1282.1亿元、增长27.8%；11项主要经济指标增速位居全省首位。重点工作取得新进展，宿淮铁路宿迁段建成通车，运河中心港开港运行，宿迁和沭阳开发区升格为国家级开发区，行政审批制度改革、徐宿淮盐铁路争取、综合保税区创建、社会信用体系建设等事关宿迁长远发展的重点工作，取得了阶段性成果。民计民生实现新改善，各级财政用于民生的投入达195.4亿元，占公共财政支出的65.1%；城乡居民收入连续10年保持两位数增长，农民人均纯收入首次突破万元；全市小康建设25项指标中有21项达标，宿豫、沭阳有望率先通过小康考核验收。

一是突出抓好产业扩量提质，转型升级取得积极进展。

工业经济质态快速提升。扎实推进两访两帮、服务中小企业十大行动等主题活动，出台大企业（集团）培育、工业再造、技术改造等政策意见，规模以上工业企业净增525户、累计达2547户，完成增加值730亿元、增长18.3%；销售超亿元企业净增153户、累计达513户。深入推进千企升级行动计划，四大传统产业实现销售1600亿元，占规模以上工业的60.6%。全面启动六大新兴产业集聚区建设，加快培育绿色建材产业、白色家电产业，新兴产业实现销售610亿元、增长35.6%。高新技术产业实现产值520亿元，占规模以上工业比重提高到19%。薄膜材料基地被认定为国家火炬特色产业基地，洋河新区获批筹建全国白酒知名品牌示范区。服务业发展提速升级。完成增加值655亿元、增长13%，增速四年来首次高于地区生产总值增长。旅游业总收入突破100亿元，接待国内外游客突破1000万人次，其中市区接待团队游客超过50万人次。物流总费用占地区生产总值比重下降到18.5%。软件与服务外包产业主营业务收入突破40亿元。现代农业规模不断扩张。深入推进水稻“四改”工程，粮食生产实现“十连丰”。新增设施农业10.5万亩、渔业2.7万亩，新增调整市级农业龙头企业88家、省级17家，生猪大中型规模养殖比重、无公害水产品产地面积比重位居全省第一。获得夏季秸秆禁烧和综合利用工作全省先进地区称号。科技创新能力明显增强。发放科技创新券4747万元，惠及533家中小企业。新增省级企业研发机构41个，大中型工业企业和规模以上高新技术企业实现研发机构全覆盖。苏北工业技术研究院获省立项支持，8个项目获国家火炬计划立项，专利申请量增幅连续两年位居全省首位，再次荣获全国科技进步考核先进市称号。实施“宿迁英才”计划，新引进国家“千人计划”专家10名，全市人才资源总量达34.7万人。

二是深入实施外向带动战略，开放型经济实现重大突破。

招商引资成效明显。创新招商体制机制，建立“四新”重大项目月度公示制，组建央企及重大项目招商办，成功举办第三届经洽会、央企合作推进会、首届中国绿色建材产业合作论坛等活动，新引进亿元以上项目355个、新开工244个、新竣工177个，竣工项目完成固定资产投资611.7亿元。对外开放水平提升。实施招引千家台企“相知行动”，精心策划“台湾宿迁周”活动，深耕精耕台湾，再次获得台商投资“值得推荐城市”称号。实际到账外资5.18亿美元，完成进出口总额31亿美元。成功举办世界华校校长、驻外使节、德国北威州议会代表团宿迁行等外事侨务活动，与新西兰因弗卡吉尔市建立国际友城关系。平台建设创新突破。探索实施“港站快速转场/集港通关”模式，启动综保区创建工作。宿迁及沭阳、泗阳三家开发区进入全省开发区综合考评第一板块，泗洪、宿城开发区分别提升21位和2位；宿迁高新区创建国家级高新区取得积极进展；6个共建园区全部进入全省第一方阵，苏宿园区连续四年位居第一。

三是加快完善城市功能设施，城镇建设迈出更大步伐。

城镇空间布局优化提升。制定实施“129”城镇发展战略，九个小城市顺利挂牌并完成总体规划编制。重大项目建设快速推进。深入实施中心城市163项重点基础设施项目，加快核心区建设，宿迁1897、雨润广场等城市综合体项目主体完工，克拉嗨谷、嬉戏谷动漫王国等旅游业项目开放运营，钟吾国际学校、市人民医院新外科大楼等公共服务项目建成使用，环城西路改造、发展大道南延至洋河段等交通工程竣工通车，分淮入沂整治及500千伏龙湖、沭阳、宿豫东3座变电站等重点项目顺利实施，南水北调东线宿迁境内工程全线通水。市政基础设施持续完善。5个老旧小区改造顺利完成，48个老小区燃气改造竣工投用，62个公交站台改造投入使用，市区新增机动车泊位2.7万个、非机动车泊位90万个，更换交通护栏2万米，新建改建公厕267座。沭阳、泗阳、泗洪三县城区面貌发生新的变化。城市管理合力逐步加强。统筹推进“三创联动”，创卫通过国家暗访，创模通过省级预评估，创建省级文明城市通过城市文明程度指数测评暗访。荣获省节水型城市称号。泗阳县创成国家卫生县城。成立市城管委，立下七个方面“宿迁规矩”，大力开展城市环境综合整治行动，城区环境和秩序显著改观。生态文明建设成效突出。研究出台“1+5”生态文明建设制度，实施市区空气质量提升四项行动，制定《全民生态文明教育规划》，化工园区和重点企业治理取得积极进展。新创省级生态乡镇16个，新增植树造林面积8.6万亩，荣获全国绿化模范城市称号。

四是扎实推进重点领域改革，内生动力有了明显增强。

行政审批制度改革加快推进。制定“1+10”改革体系，突出工商登记、“四证（照）一体”登记等方面的流程再造和创新，市级审批事项仅保留57项，精简率超过70%，不仅有力推动了政府职能转变，而且有效激发了市场主体活力。全年新增个体工商户9万户、私营企业1.77万户，分别增长37.9%、154.3%。土地管理创新稳步推进。完善地票交易制度，加快城乡统筹土地管理创新试验区和泗洪西南岗地区统筹城乡发展路径创新实验区建设，农村土地确权发证、“双置换”、城乡建设用地增减挂钩等工作顺利开展。投融资体制改革深入推进。扎实做好直接融资，短期融资券、区域集优融资实现突破，全年新增直接融资105亿元。积极推进平台实体化运作，市属5家投资公司资产总额达841.1亿元。

五是稳步提升公共服务水平，惠民实效得以充分显现。

创业就业工作成效明显。开展创业培训1.51万人次，实现创业带动就业4万余人。新转移农村劳动力5.3万人，城镇新增就业4.98万人、再就业2.68万人，城镇登记失业率2.38%。社会保障水平显著提升。全市“五险”扩面26万人次，城镇居民医保、养老参保率分别达98%和100%；城乡低保标准提高到每人每月350元和270元，五保集中和分散供养标准提高到每人每年5100元和4300元；新开工各类保障性住房5万余套、基本建成3.4万套，完成棚户区改造266万平方米、农村草危房改造1万余户。扶贫开发取得积极进展。稳步推进泗洪西南岗、成子湖周边和黄墩湖滞洪区等三大片区扶贫开发，加大黄河故道地区农业综合开发力度，机关单位干部职工与低收入农户“一对一”结对帮扶实现全覆盖。各项社会事业协调发展。新增省优质园39所、省三星级高中1所，高考本科达线人数连续四年超万人、清华北大录取人数跃居全省第二；顺利完成中心城区教育布局调整和学校管理体制调整。市第一人民医院全面封顶，市心血管医院挂牌运营，宿迁、沭阳中医院分别创成三甲和三乙中医院；基本药物、新农合结报实现村级全覆盖，政策范围内住院补偿比例提高到75%，老百姓用药负担大大减轻。人口计生工作通过省“十二五”中期评估。市档案馆、图书馆、博物馆建设扎实推进，乡镇综合文化站、农家书屋、社区文化中心功能不断完善。成功举办首届骆马湖生态四项公开赛。全面掀起学习张森热潮，宿迁英雄、好人群体效应释放出强大正能量。民防、物价、气象、食品药品安全、民族宗教、人民武装和双拥、档案、党史、地方志等工作实现新进展，妇女、儿童、青少年、老龄、残疾人、红十字、慈善等事业取得新进步。社会管理创新不断加强。扎实推进平安创建、综合治理、信访维稳等工作，深入开展打黑除恶、犬类整治、交通秩序治理等专项行动，公众安全感和社会治安满意度位居全省前列。

六是高度重视民主和法治建设，政务环境持续优化提升。

坚持把抓落实、办实事作为政府工作的生命线，对日常工作每周一会商、重点项目每月一会办、经济形势每季一分析。深入开展“三解三促”活动，广泛开展“民声”大征集、“群众满意的机关服务品牌”创建活动。便民方舟功能进一步优化，政务服务效能显著提升，12345政府热线服务平台建设取得实效。成功举办中国政务微博路在何方高层论坛，“网上宿迁”跻身中国政府网站绩效评估前四强。扎实推进诚信宿迁网、企业信用数据库、重点人群信用数据库建设，社会信用体系框架初步形成。认真落实中央《八项规定》、省委《十项规定》和市委《实施意见》，完善规范性文件制订公开征求意见、常态化听证、专业性评价等制度，构建法治政府建设“1+2”考核制度体系，依法行政和廉洁从政水平不断提升。“六五”普法通过省中期考核验收。自觉接受市人大及其常委会的法律监督、工作监督和市政协的民主监督，主动执行市人大及其常委会决议，全面完成人大代表议案、政协提案办理任务。

各位代表，过去一年，在宏观环境复杂严峻的形势下，我市经济社会发展之所以取得良好业绩，离不开省委、省政府和市委的正确领导，离不开省级机关和苏州等兄弟市的关心帮助，离不开人大、政协和社会各界的监督支持，离不开全市人民的团结一心、奋力拼搏。在此，我代表市人民政府，向奋战在各行各业的全体劳动者，向人大代表、政协委员、各民主党派、工商联和无党派人士、各人民团体，向离退休老同志，向中央和省驻宿单位以及人民解放军、武警官兵、公安干警，向参与宿迁建设的海内外客商、高层次人才、广大援宿干部，以及所有关心支持宿迁发展的各级领导、各界人士，致以崇高的敬意和衷心的感谢！

在肯定成绩的同时，我们也清醒地看到，全市经济社会发展中还存在一些矛盾和问题：经济发展方式有待转变，产业结构仍不合理，转方式调结构的任务仍然繁重；城乡统筹步伐有待加快，城镇化质量依然不高，中心城市首位度亟待提升；社会管理创新有待强化，社会组织发育仍不充分，公平竞争环境仍需优化；民生事业建设有待加强，部分群众生活还比较困难，公众知情权、选择权、表达权、监督权等尚未完全落实；政府管理职能有待改进，少数部门服务发展能力不足，服务意识、行政效能尚需进一步强化。对于这些问题，我们将采取有效措施，认真加以解决。

2014年目标任务

今年是贯彻落实党的十八届三中全会精神、全面深化改革的第一年，也是攻坚“十二五”、建设小康社会的关键之年。当前，世界经济仍处于深度调整之中，我国经济也处在增长速度换挡期、结构调整阵痛期和前期刺激政策消化期“三期”叠加的特殊阶段。在增强忧患意识、坚持底线思维的同时，我们也充分认识到，我国经济发展长期向好的基本面没有变，宿迁发展面临着诸多机遇和有利条件。体现在改革之利上，十八届三中全会作出了全面深化改革的战略部署，释放出海量的改革信息和制度红利。宿迁拥有改革的浓厚氛围和群众基础，在土地管理、行政审批等关键性改革上抢占了先机，只要顺时应势、借势发力，改革的优势必能转化为发展的胜势。体现在政策之利上，中央陆续出台了一系列经济金融领域的重大政策，省委、省政府专门制定了支持苏北地区全面小康建设的28条意见，加大了对宿迁的特殊扶持力度，只要找到结合点、找准对接点，定能将上级的普惠性政策为我所用，把支持宿迁的特殊性政策变为加快发展的红利。体现在成长之利上，宿迁发展已经步入快车道，即使面对各种风险挑战，主要经济指标依然保持了较快增长，加之正处在工业化、城镇化的快速推进期，发展的空间很大，成长的动力很足。只要我们保持稳的定力、好的导向、快的标准、干的士气，善借改革之利、巧借政策之利、用好成长之利，就一定能够开创宿迁科学发展、跨越发展、和谐发展的新局面。

今年政府工作的总体要求是：全面贯彻落实党的十八大、十八届三中全会、省委十二届六次全会、中央和全省经济工作会议精神，以改革创新统揽经济社会发展全局，紧紧围绕建设小康社会的奋斗目标，按照市委四届四次全体（扩大）会议要求，坚持稳中求进工作总基调，深入开展统筹“三大发展”、推进“九比竞赛”活动，加快转变经济发展方式、城乡发展路径、社会治理模式、政府管理职能、民生服务机制，推动经济社会又好又快发展。

经济社会发展的主要预期目标为：地区生产总值增长12%左右，公共财政收入增长15%左右，固定资产投资增长23%左右，社会消费品零售总额增长14%左右，进出口总额增长8%左右，实际到账外资增长10%左右，城乡居民收入均增长12%左右，节能减排完成年度目标任务。

实现上述目标，必须坚持改革为先，以重大问题为导向，不贴标签，大胆假设，小心求证，将改革发展中遇到的问题变为议题，从解决一个一个议题入手推动改革的进程，以先行先试增创竞争优势，以创新突破赢得发展先机。坚持发展为要，千方百计上项目，全力以赴增投入，集中精力做产业，既力求吃得饱，又力争跑得快，做到“换挡”不降档，以发展速度的持续加快和领先，推动总量规模的逐步进位和赶超。坚持生态为重，更严标准实施项目审批，更强决心推进硬件建设，更硬手段加强污染治理，更高要求保护土地资源，绝不以牺牲生态环境为代价追求一时的经济增长，给子孙后代留下天蓝、地绿、水净的美好家园。坚持民生为本，遵循尽力而为、量力而行的原则，办妥办实一批顺民意、解民忧、保民安的好事，更加注重权利公平、机会公平、规则公平，真正让人民群众成为幸福宿迁的建设者和共享者。

新的一年，我们将坚定不移做好八个方面的工作：

一、坚定不移推进改革创新，激活发展内生动力

宿迁与其它地区相比，能源不是优势、资源不是优势、基础更不是优势，惟有改革创新是最大优势。我们将坚持以发展中的问题和群众反映的难题为导向，拿出更大的智慧、勇气和坚持，全面落实好市委、市政府《关于近两年深化改革的若干要点》，充分尊重群众和基层的首创精神，鼓励先行先试、探索创新、积累经验，齐声唱响宿迁改革创新的主旋律，努力续写宿迁改革创新的新篇章，真正让改革创新成为我们的习惯。

推进市场化改革。正确把握政府与市场的关系，政府主动从竞争性领域退出，最大限度减少对微观经济事务的干预，该交给市场的交给市场，该交给社会的交给社会，真正让市场在资源配置中起决定性作用。深化行政审批制度改革，动态调整和精简审批事项，完善和扩大并联审批，推进多证（照）合一，逐步实现网上公开透明运行，让企业和群众少跑路、更便捷；探索推进资格资质脱政化改革，通过委托、授权等方式，逐步将资格资质培训、认证权力和职能从政府部门剥离出来、推向社会；创新对市场主体的监管方式，推动一次性审批向全过程监管转变。实施政府机构改革与事业单位分类改革，调整计生、卫生、食品安全监管等机构和职能。加大政府购买服务力度，研究制订政府购买服务“负面清单”，除明确不能购买的事项外，其它全部向市场购买服务，争取在三年内实施到位，让市场主体更多更好分享到改革创造的商机。探索市属权限内资源要素价格市场化改革，积极开展大用电客户与发电企业直接交易试点，推行排污权有偿使用和公开交易试点，让企业和群众实实在在享受到改革带来的实惠。

深化社会事业领域改革。围绕解决人民群众反映强烈的“看病难、看病贵”问题，深化医药卫生体制改革，巩固医疗改革成果，激活各类医疗机构的发展活力。规范和完善基本药物制度，降低广大群众看病买药费用；研究制订加快推进社会资本举办医疗机构的意见，创造公平竞争环境，促进医疗市场充分良性竞争；加大对民营医院的扶持力度，逐步扩大以奖代补基金的规模，考核和引导民营医院更好地承担公共医疗服务；创新机制强化对民营医院的监管，以制度化的规则推动其依法依规经营、持续健康发展，让病患享受愈来愈好的医疗服务；建立医护人才引进与激励机制，确保引得进、留得住、用得好，提高医疗机构的发展与服务水平。围绕激活民办教育发展活力，创新民办教育发展机制，发挥扶持政策杠杆作用，撬动更多民资注入教育领域；鼓励采取组建教育集团等形式，优化重组民办教育机构，提升民办学校办学水平。围绕打造文化人才集聚和文化产业发展新高地，探索柔性引才机制，建立以政府奖励为导向的人才激励机制，努力培育一批在全省全国有影响的文学、书画、演艺领军人才；鼓励文化企业和社会力量共同参与文化创作、文化产品的供给，加快创作一批体现宿迁地域特色、具有较高水准的文化艺术精品，打造宿迁新品牌，树立宿迁新形象。

探索农村综合制度改革。围绕激活农村发展活力，增加农民财产性收入，推进以产权制度改革为核心的农村综合改革。健全农村产权权益实现机制，推进集体建设用地、宅基地使用权确权登记，建立市、县、乡三级农村产权交易综合平台，让农村居民与城市居民一样实现资源资产的保值增值。完善土地流转制度，坚持规划管控和用途管制，探索设立农村“土地银行”，吸储农民自愿流转的零散土地，以“零存整贷”的方式推动农业规模化经营。建立农村产权保护机制，在市、县（区）建立农村产权维护援助中心，成立市农村产权仲裁院，推动农村产权流转交易公开、公正、规范运行。加快推进城乡统筹试验区建设，以农民生产方式变革引领生活方式转变，让农民融入城镇、幸福生活。

二、坚定不移推进新型工业化，壮大特色优势产业

宿迁今天所有问题的根源在于发展不充分，最主要的原因是工业化水平不高。特定的发展阶段决定了我们必须坚定不移地走新型工业化道路，以工业总量的扩张和产业质态的提升，创造更多更好的财源税源，创造更多更好的就业岗位，创造更多更好的增收渠道，实现经济发展与民生幸福的同频共进。

深化招商引资，扩大工业总量。坚持全员招商与专业招商双轨并行，强化经济主管部门、相关职能部门和各类招商主体作用，大力开展产业招商、主题招商，集中精兵强将主攻重特大项目，全力在产业链条关键环节和核心项目的招引上实现新突破。进一步优化以市场换投资、资源换资本以及支持企业拓展市场等方式，再造招商新优势，吸引更多企业入驻宿迁。强化项目的研究、策划、设计和争取工作，注重与国内外知名产业研究院、专业咨询公司等机构合作，力求在招引世界500强企业、跨国公司中把握先机、谋求突破。建立健全招商活动综合评估机制、差别化的政策支持和项目评估审定机制，提高招商针对性和项目含金量。

加速转型升级，提升产业质态。研究制订差异化的激励政策，重点支持大企业（集团）培育计划中的骨干企业、市场占有率高的行业龙头企业、走专业化差异化道路的特色企业、在本地能构成相对完整产业链的关键企业做大做强、转型发展。大力实施新兴产业千亿计划，突破一批核心项目，引导企业参与技术标准制订，年内实现销售800亿元。聚焦支持六大新兴产业集聚区建设，推动光电和智能电网产业实现新的突破。深入实施千企升级行动计划、工业强市六大行动和工业再造计划，加快四大传统产业提档升级，年内销售突破2000亿元。借助中国绿色建材产业合作论坛，实施绿色建材强制推广和应用工程，打造国家级绿色建材产业先导区、集聚发展示范区和绿色建材市场集中区。推动环保新材料、精密仪器及关联产业发展。以双鹿上菱冰箱项目产品下线为契机，发展空调、冰箱冰柜、洗衣机等家电产业，做好格力电器、东贝压缩机、人民电器等项目建设服务工作，吸引冷凝管、压缩机等机械零部件、电子元器件的配套企业集聚，打造国家级白色家电节能环保配套产品供应基地。

持续优化环境，助推企业发展。坚持稳企业就是稳增长、稳运行就是稳岗位，进一步优化企业发展环境，强化要素保障供给，让广大企业家安心生产、放心经营。创新工业经济运行监测分析方式，加强对苗头性、倾向性问题的研判，全面了解重点行业、重点企业生产经营状况，帮助企业把握发展走势、掌握市场主动。认真做好资金、水、电、气、劳动力等生产要素调度，支持有订单、有效益的工业企业开足马力生产，提高工业有效生产和销售。扎实推进两访两帮活动，定期召开工业企业座谈会，完善领导挂钩联系、分级会办、工作督查三项制度，优化问题会办、交办、反馈机制，为困难企业雪中送炭，助推企业爬坡过坎、度过难关。

三、坚定不移推进现代农业，转变农业生产经营方式

宿迁是传统农业大市，农民是人口的主体，任何时候都不能忘了土地，任何阶段都不能偏废农业，任何时期都不能亏待农民。做好新时期“三农”工作关键不是要命令农民做什么，而是不要轻易禁止农民做什么。我们将遵循现代农业发展的方向，充分尊重广大农民的意愿，加快转变农业生产经营方式，以最少的人种最多的地，努力在减少农民中富裕农民。

推进现代农业发展，提升农业产业化水平。按照“县区有特色、乡村有特品”的要求，加快发展特色农业，做精区域特色板块。加强现代农渔业产业园区建设，引导省级园区转型发展，年内新增设施农业10万亩、渔业2万亩。扎实推进生态农业和放心粮油工程建设，逐步建立从田间到餐桌的全过程监管机制，保证舌尖上的安全。围绕产业链前端的种子产业和后端的加工产业，培育和招引一批有品牌、有实力的龙头企业，年内新培育销售2000万元以上龙头企业20家。加大对市农科院、南农大（宿迁）设施园艺研究院支持力度，推动产中服务逐步向产前和产后技术推广深化，不断强化农业“三新”成果推广应用。积极引导经营性主体发展专业化服务，完善基层农技推广服务体系。组织开展各类农民培训，培育一批懂技术、善管理、会经营的新型农民。

培育农业规模经营主体，构建新型农业经营体系。鼓励和支持农民在实践中探索机制灵活、形式多样的农业生产经营方式，培育由市场自发形成的合作经济组织，帮助更多农民走上合作致富之路。加大对专业大户和家庭农场的扶持力度，助推更多农民走上经营致富之路。鼓励多种主体参与土地股份合作社，开展村级土地股份合作社试点，引导更多农民走上入股致富之路。深入开展“五好”示范社创建活动，不断提高农民专业合作社规范化管理水平，强化其在农资购买、农产品销售、农机作业等生产服务方面的作用，提供更加便捷优质的产前、产中、产后服务。

加强农村基础设施建设，改善农民生产生活条件。实施农田水利提升工程，加快大型灌区续建配套、农村防洪排涝等工程建设，提高小型农田水利工程管护标准化水平，打通“毛细血管”，解决好“最后1公里”的问题，真正实现遇旱不慌、临雨不急。实施农村公路提档工程，提高桥梁、道路等基础设施建设标准，年内新建改建农村公路道路1019公里、桥梁155座，完成路肩硬化68公里。实施镇村公交通达工程，年内新增通车乡镇10个，全市镇村公交开通率达60%，逐步解决农村居民“出行难”问题。加快实施城乡区域供水工程，年内新增通水乡镇26个，区域供水覆盖率提高到86.2%，再让78.9万城乡居民喝上放心水。

四、坚定不移推进第三产业，提升现代服务业发展水平

服务业不仅直接关系和影响到实体经济的发展，而且具有集聚人气、吸纳就业、带动消费、便利生活的能力。我们将坚持生产性服务业与生活性服务业并重，深入实施服务业“3119”工程，完善现代服务业体系，满足人民群众不同层次的消费需求。

建设省级现代服务业集聚区。围绕打造现代服务业发展新高地，优化发展教育培训业，推动现有职业院校整合资源、突显特色；借助事业单位和社会机构力量，建设培训、人事考试和鉴定发证“三位一体”平台，促进法律、会计、评估、人力资源等商务服务业企业向综合化、规模化方向发展，建成全国知名的资格资质认证、培训产业基地。培育发展健康服务业，依托骆马湖省级旅游度假区，规划建设康复疗养基地，发展养老服务、康复疗养等产业；结合三台山森林公园扩面提质，打造集休憩、疗养、健身为一体的高端养生场所。积极发展文化产业，加快运河特色文化基地建设，年内创成国家级文化旅游示范区；建设三台山文化创作基地，打造集学习、培训、展览等为一体的琴棋书画培训中心。加快发展总部经济，重点建设一批项目承载基地，引进总部经济企业入驻，打造国内知名的生态型总部企业集聚区。

繁荣幸福路和霸王举鼎两大商圈。加快建设宿迁1897、雨润广场等重点商业设施，启动国泰广场改造，年内水韵城建成运营，中豪国际广场竣工验收。集中建设核心商圈，邀请国内外著名规划和商业专家“把脉问诊”，提高楚街、宝龙24街、金鹰滨水特色休闲街等现有商业设施利用率，推进商业商务中心人气集聚和业态繁荣，真正实现消费在家门口、税收留在宿迁。

发展旅游、物流和软件服务外包三大产业。围绕建设“玩不够”的城市，启动旅游业新三年提升计划，加快推进项王故里景区三期、三台山森林公园扩面提质工程等景区项目建设，完善提升克拉嗨谷、嬉戏谷动漫王国等旅游景点；统筹策划各类节庆，承办环骆马湖生态四项国际公开赛，支持社会力量和企业办好湖鲜美食节、骆马湖开渔节等主题节庆活动；做好团队游的组织和落实工作，年内实现旅游总收入130亿元，接待国内外游客1300万人次，其中市区接待团队游客突破80万人次。加大运河中心港产业园建设力度，突破传化公路港等一批重点物流业项目，积极招引第三方物流龙头企业和制造业龙头企业自身的物流项目，支持通连国际物流公司开展甩挂运输试点，进一步降低企业物流成本，年内物流总费用占GDP比重降至18%左右。加快推进京东商城华东云计算中心及研发中心、德俊数码产业园等项目建设，培育招引一批标志性、引领性的电商企业，争创省级服务外包示范城市和更多省级电子商务基地，年内软件和服务外包产业主营业务收入突破50亿元。

五、坚定不移推进开放型经济，加快经济国际化步伐

发展开放型经济不仅有利于提升区域开放水平、促进经济快速增长和转型升级，而且有利于打造符合国际惯例的商务环境、政务环境、生活环境，同时对于开阔人的视野、提升人的眼界都具有重要的推动作用。我们将坚持外向带动战略不动摇，更加广泛地拓展开放领域，更大范围地集聚要素资源，提升开放型经济发展水平。

拓宽对外开放领域。以台资、港资、德资为重点，全力主攻外资大项目。深入开展“相知行动”系列活动，依托宿迁经开区台商创业创新科技产业园和各县（区）台商集聚平台，力争3—5年集聚2000家台资企业。实施外贸总量扩张计划，促进贸易便利化，推动异地进出口转化。引导企业参加各类展会、开拓新兴市场，年内新培育进出口企业100家、出口超千万美元企业5家。支持企业“走出去”，加大国际市场开拓力度。进一步拓展对外交往格局，深化与国际友城友好关系，在德国、西班牙等国发展产业匹配度较高的新友城，构筑全方位、宽领域、多层次对外开放格局，让宿迁走向世界，让世界走进宿迁。

提升区域合作水平。以感情为基础、利益为导向，创新南北挂钩合作机制，发挥市承接产业转移办公室作用，探索与苏州建立产业有序转移、利益合理分成的新机制，打造全省新型纺织和机械电子类产业转移重点承接地。认真研究对接上海自贸区，强化对自贸区产业信息的收集、研判，积极招引溢出的先进制造业、服务外包产业和生产性服务业项目。加强淮海经济区沟通合作，推动交通、市场、社保、环保等一体化进程，促进旅游线路互联互动、客源互享互送、营销互利互惠，实现区域合作共赢。

建设高端平台载体。按照“申报、建设、项目落户、服务监管”同步推进的思路，加大综保区争取和建设力度，上半年具备申请国家部委验收条件并力争早日获批，打造招商引资和开放型经济发展的新平台。围绕建设永不转移的产业园，宿迁经开区加快先进制造业项目集聚，提升基础设施承载能级，年内进入全省开发区前50强，早日建成区域性开放型经济高地；沭阳经开区抓住升格为国家级开发区的机遇，加快突破一批大项目和外资项目；泗阳、泗洪、宿城开发区更加注重项目集聚和功能提升，向国家级开发区的目标迈进；宿迁高新区重点推进“一院三中心”四大功能区建设，年内基本具备国家级高新区申创条件；苏宿园区实施新三年提升计划，继续当好全省共建园区排头兵；其它共建园区同步推进招商引资和基础配套，保持全省第一方阵。

增强要素集聚能力。围绕再造人力资源优势，落实大企业用工服务机制，出台鼓励外来务工人员购房居住政策，吸引更多市外劳动力到宿迁务工。围绕再造用地保障优势，健全国有土地市场化供应制度，推进工业用地出让弹性年期和租赁制，完善地票制度，提高城乡建设用地增减挂钩周转指标使用效率。围绕再造融资供给优势，大力推进招商引行，力争南京银行、江苏长江商业银行宿迁分行上半年开业；鼓励各金融机构到基层设立分支机构、拓展业务，创造条件支持民丰银行到三县设立分支机构；积极争取信贷规模，调优信贷结构，年内新增贷款突破300亿元，工业贷款占比超过30%；引导科技型、成长型中小企业到“新三板”，以及区域性股权交易中心等场外市场融资。围绕再造科技人才支撑优势，加快“一城、四院、六中心”等创新载体和服务平台建设，发挥创新券引导作用，推动科技与金融深度融合，年内全社会研发投入占GDP比重达1.35%；大力推进“宿迁英才”计划，重点抓好高层次领军人才、高技能实用人才两支队伍，加快高层次人才创业园、产业技术研究院、企业研发机构三大平台建设，实施领军人才引进、政产学研人才开发、名校优生引进和名医名家集聚四大工程，争取省派科技镇长团三县两区全覆盖。

六、坚定不移推进中心城市建设，统筹城乡发展

宿迁城镇化的阶段性特征非常明显，中心城市基础功能配套、人气商气集聚、辐射带动能力等尚不能满足发展需要，镇村又普遍存在基础设施薄弱、产业发展不足、人口吸附力不强等问题。对此，我们将坚定不移地实施“129”城镇发展战略，进一步提升首位度、打通中心轴、建设小城市、创设新载体、构筑大交通，迅速掀起城镇建设大推进、大开发、大建设的高潮。

提升首位度。顺应中心城市发展的形势变化，建立健全“五规融合”新机制，推进财力分成、事务分担、利益分享，形成规划建设合力。围绕提高建成率，以落实核心区建设三年行动计划为抓手，加快实施180项重点基础设施项目，集中推进旧城改造，统筹各组团内部重大功能板块建设，让我们的城市内聚外联、融合发展。围绕提高配套率，在教育设施上，启动市钟吾初中改造，完成宿豫实验高中改造扩建，马陵高中新校区、宿迁经开区初中及黄河小学建成使用；在医疗设施上，全面建成市第一人民医院并实现高起点、高水平运营，市妇产医院、工人医院新院建成运营；在文化体育设施上，开工建设广播电视发射塔，完成城市记忆广场主体工程，市博物馆、图书馆、文化馆投入使用，奥体中心体育竞赛场、国际会展中心基本建成；在市政设施上，重点推进小餐饮集中区、停车设施、标准化菜市场、公交站场、公共自行车服务系统等功能设施建设，完善公厕、果壳箱、垃圾转运站、餐厨垃圾处理场等环卫设施建设，加强供电、供热、供气、供排水等基础设施建设，让我们的城市功能更加完备、生活舒适便捷。围绕提高达标率，深化“三创联动”，年内创成国家卫生城市、国家环保模范城市和省级文明城市；统筹推进智慧城市建设；完善“一街三方”联勤共管的“三位一体”机制，集中治理城市管理十大焦点难题，巩固提升城区道路交通秩序综合整治成效，创新不文明行为惩诫机制，推动“宿迁规矩”向“宿迁习惯”转变，让我们的城市更加文明、更加和谐。全面实施城市绿化景观和主要道路街景整治提升工程，打造林荫城市、彩色城市、花园城市、个性城市；加快实施河湖水系沟通提升工程，实现中心城市内环水系水上畅游目标，让我们的城市水绿交融、充满灵性。

打通中心轴。把交通建设作为中心轴的重中之重，加快省道347宿迁段、通港大道、发展大道东延等主干道建设，启动城市轨道交通前期工作，把中心城区、洋河新区、泗阳城区串成一线，辐射带动沭阳、泗洪两县发展。推进轴内规划、产业布局、重大基础设施建设联审、联建、联管，带动和激活轴内片区以及沿线乡镇的发展。加快三大节点及沿轴乡镇水、电、气、污水管网等基础设施项目建设，力争早见成效，形成局部聚合。

建设小城市。将九个小城市建设摆在突出位置，更加注重小城市全域规划与经济发展规划、各类专项规划衔接，塑造鲜明的特色和个性。以老镇区改造为突破口，统筹推进路网、管网以及医院、学校等项目建设，年内小城市建成区综合配套率达60%以上。按照“工业向园区集中、农民向镇区集中、土地向大户集中”的原则，积极探索农村人口就近市民化、就近城镇化新路径。支持和引导其它小城镇发挥优势、错位发展，走出各具特色的镇域经济发展之路。

创设新载体。在城市社区和开发区，稳步推进邻里中心建设模式，满足和方便城市居民、务工人员生活需求，让邻里关系更和睦、更融洽。在九个小城市和相关乡镇，结合农民集中居住区建设，试点建设一批集公共活动室、健身场所、阅览室、小超市、卫生室、幼儿园、红白事理事堂、车站停靠点等为一体的乡里中心，以及满足一家一户基本种植养殖需求的公租农庄，让乡里中心成为方便村民生活、滋养乡情记忆的场所，让乡村成为久居的人感恩、远行的人牵挂的地方。

构筑大交通。立足宿迁长远，增强发展后劲，加大重点交通基础设施项目争取力度，协调跟进徐宿淮盐铁路项目，做好征地拆迁等前期工作，年内开工建设；对接争取通用机场项目，提前启动项目建议书和可研编制，争取早日开工建设；加快推进运河中心港基础设施建设，年内力争获批二类口岸；加大国省干线道路建设力度，推进省道121、省道326、省道245苏皖省界至泗洪段、成子河船闸等项目续建工程，启动宿扬高速宿迁段、省道329、省道330溧河洼大桥及接线等项目工程，着力构建公铁水空一体的现代化综合交通运输体系。

七、坚定不移推进社会事业发展，满足人民群众公共需求

提供公共服务，满足公共需求，是现代政府的一项基本职责，也是衡量政府管理水平和能力的重要标准。我们将以人民满意为标杆，加快发展社会事业，增加公共产品供给，为人民群众提供更方便、更优质、更均衡的公共服务。

促进卫计事业提升发展。着力健全覆盖城乡的医疗卫生体系，积极引进全国知名医疗机构，合作建设高档次健康体检和康复中心，新建市公共卫生服务中心；结合小城市发展布局，规划建设区域性公共医疗卫生中心；加快村卫生室标准化建设，推进新建卫生室与乡里中心有机融合。进一步优化配置医疗卫生资源，鼓励各级各类医疗机构提升办医层次，支持市人民医院争创三甲，年内乡镇医院30%以上达到一级甲等标准，40%以上达到一级乙等标准。创新卫生监督和新农合监管机制，规范医疗卫生服务行为。加强人口和计划生育工作，做好“单独两孩”生育政策专项调查并适时实施，推进幸福家庭创建活动，稳定低生育水平，提高出生人口素质。

促进教育优质均衡发展。以教育现代化和全国义务教育发展基本均衡县（区）创建为抓手，全面提升宿迁教育事业发展水平，努力让所有孩子都能接受良好教育，站在公平的人生起跑线上。加快发展学前教育，实施学前教育五年行动计划，新建改建幼儿园120所，创建省优质园25所；均衡发展义务教育，优化城乡教育资源配置，改善农村薄弱学校办学条件；夯实做强高中教育，支持薄弱高中创建三星级高中，推行热点普通高中招生指标均衡分配政策；稳步推进高等教育，积极引进市外优质高教资源，推动高师、经贸、卫校建设独立设置的专科院校，争取年内宿迁学院申本成功；全面提升职业教育，加快专业设置调整，建立“学校+工厂”、“授课+实践”的培训模式，年内职校毕业生“双证率”达80%，当年一次性就业率达95%。高度重视教师队伍建设，建立名师培养目标和梯队建设规划，着力打造一支结构合理、师德高尚、专业水平高、教学能力强的优秀教师队伍。启动名师牵手农村教育工程，组织优秀教师到农村交流支教。

促进文体事业繁荣发展。大力实施文化惠民工程，加快县（区）文化场馆、社区文化中心、农村文化设施建设，免费开放公共文化设施。扎实推进有线电视进村入户工程，进村入户率提高到85%以上，让农村居民看上更加丰富多彩的节目。加强基本公共体育服务体系建设，加快建设“城市社区十分钟健身圈”，积极开展群众性健身活动，打造一批具有影响力的品牌赛事。

八、坚定不移推进民生改善，提高人民群众生活水平

解决民生问题是最大的政治，改善民生福祉是最大的政绩。我们将怀揣真挚的百姓情怀，秉承“民生为大”的理念，深入实施民生改善“一六二一”工程，把更多财力物力向民生领域倾斜，下决心做好普惠性民生、特殊性民生、基础性民生、增长性民生，在老百姓少跑路、少负担、少疑虑、少烦心上多下功夫，让公众在细微之处和点滴变化中真切感受到党委政府的温馨与敬意。

在加强创业就业中富民。全面落实全民创业三年行动计划，强化创业培训、创业实训和创业孵化，年内新建创业孵化基地5个。加大金融、财税政策支持力度，重点扶持城乡劳动者初次创业和中小微企业发展，引导培育与市内工业相配套的全民创业项目，年内扶持成功创业1万人，通过省级创业型城市验收。大力开展“春风行动”，加强技能培训，开发公益性岗位，加快发展建筑业、服务业等富民产业，年内城镇新增就业2.5万人，帮助1.2万名失业人员实现再就业。加大扶贫开发力度，推进三大集中连片贫困地区扶贫开发和黄河故道地区农业综合开发，落实整体帮扶规划和扶贫菜单，年内全市累计75%的农村低收入人口实现脱贫、75%的经济薄弱村实现新八有目标，完成2804户农村草危房改造和省下达的农村危房改造任务。

在提高保障水平中利民。牢记保障线就是生命线，进一步完善社保体系，推进社保扩面“幸福行动”，年内企业职工“五险”扩面新增26万人次；加快建立城乡统一的居民医保制度和管理体制，开展医保异地结算互通行动，落实大病保险制度，切实减轻群众看病负担；强化被征地农民社会保障，年内参保比例达95%以上，保障好被征地农民的合法权益和长远生计；进一步强化基层服务，年内发放社保“一卡通”200万张，逐步实现参保人员人人持卡。完善房屋征收安置市场化机制，推行以市场化为主、政府统建为辅的安置方式，有效缩短过渡期，让拆迁群众早日住上心仪的房子，安居乐业、幸福生活。加快完善社会救助体系，提高各类补贴标准，优化特殊困难群众生活救助与物价补贴动态机制，做好法律援助、希望工程和困难职工援助救助工作，让困难群众生存有尊严、生计有保障、生活有盼头。

在兴办为民实事中惠民。坚持民生优先、急需先行，办好八大类81项为民办实事项目。按计划推进民生六项工程建设，年内完成15个老旧小区、160个公交站台、57个老小区燃气工程改造任务，建设改造30个“两场一街一中心”项目，完成环城西路、开发区大道等重要道路和4个城市出入口、5个桥头公园景观绿化，实现中心城区雨污分流、居民小区管网配套全覆盖。编制《宿迁市城市供热工程规划》，加快市区热源点和分布式能源点建设，同步推进工业和居民供热，让市民逐步用上安全放心的暖气。完善适度普惠型社会福利体系，建立健全政府购买养老服务制度，加强居家养老服务设施建设，改造提升农村敬老院30个，新建居家养老服务中心125个。落实高龄补贴政策，实施贫困家庭儿童重大疾病慈善救助制度，加强孤残儿童福利保障和留守儿童关爱工作。落实惠民殡葬政策，启动新殡仪馆和公墓建设。推动残疾人事业更好发展。

在建设生态文明中为民。像爱护眼睛一样呵护宿迁的生态环境，全面落实“1+5”生态文明建设制度，一手抓生态建设，一手抓污染治理，做到多还旧“账”、不欠新“账”。加快生态示范创建，年内创成国家级生态乡镇6个、省级10个，市级生态村占比达60%，泗阳县创成省级生态县。深入推进市区空气质量提升四项行动，建立大气污染防治区域协调机制；进一步加大财政投入，完善管理机制，下决心实现秸秆全面禁烧、全部清运、全量利用，让蓝天白云成为宿迁的城市品牌。加快污水处理设施和管网建设，稳步提高治污减排能力。扎实开展中小河道水环境综合治理和城区入河排污口整治，推行跨界断面生态损害资金调节制度。严格推进环境综合整治，强化化工园区治理和监管，加强重点企业环境监察，完善农村生活垃圾收集转运体系和农民集中居住区污水集中处理设施，实现污染防治设施正常运行、污染物稳定达标排放。加快耿车循环经济产业园“三集中”步伐，同步推进企业入驻、项目清理和环境整治，从根本上改善当地生产生活环境。

在创新社会治理中安民。加强社区管理创新，推进“政社联建”，实施网格化管理。深化平安宿迁建设，严厉打击各类违法犯罪活动，加快推进道路、小区等重点部位技防设施建设，构建立体式的社会治安防控体系，维护让坏人有怕头、好人有奔头的良好局面。强化安全生产责任制，建立安全生产隐患排查治理和安全预防控制体系，完善考核点评、安全生产“黑名单”制度，全力构建安全生产“责任网”、“监督网”和“保障网”。积极推进网上受理信访，完善社会矛盾纠纷大调解机制，层层落实信访工作责任，努力从源头上预防和化解信访矛盾。扎实推进“六五”普法，提高全社会法律意识和法律素质，让人民群众在法治中感受公平正义。整合各类应急资源，全面提升综合应急能力。加强社会信用体系建设，加快建立市公共信用信息平台，做好信息整合和信用管理，实现信用信息联网互通、共享共用，建立健全“守信激励、失信惩诫”的制度。充分发挥鼎鼎有民、网络问政、12345政府热线、宿迁之声、政行风热线等平台作用，整合对接各类问政平台办理渠道，形成有序高效的民意诉求收集办理机制。做好人民武装和“双拥”共建工作，进一步推进军民融合。

加强和改进政府工作

新形势新任务对政府建设提出了全新的课题，也提出了更高的要求。我们将以开展党的群众路线教育实践活动为契机，继续发扬改革创新的优良传统，着力突破传统的思维定势、行为方式和发展理念，加快转变社会治理方式，推动全能政府向有限政府转变、管制政府向服务政府转变、权力政府向责任政府转变。

以权力清单规范权力运行。按照“明晰单位权责、明晰职位权责”的要求，制订部门权力清单，推动行政权力透明公开。积极构建网上公开透明运行系统，让权力在阳光下运行，不仅将静态权力告知公众，而且将每种权力运行过程“示众”，方便公众对政府权力的监督，倒逼部门在边界内行使职权，把权力关进制度的“笼子”里，给权力涂上“防腐剂”、戴上“紧箍咒”。

以充分放权激活社会组织。将应由社会组织履行的事务性、服务性职能，全部交给各类社会组织承担，强化对中介机构的扶持、培育和监管，提高社会组织承接政府转移职能、公共服务和中介服务的能力。

改革社会组织登记制度，除依据法律法规需前置行政审批及政治法律类、民族宗教类社会组织外，全面推行“五放五减十鼓励”举措。制订出台加快推进社会组织培育发展意见，培育发展行业协会商会类、科技类、公益慈善类、城乡社区服务类社会组织，年底实现每万人拥有6个以上社会组织。

以程序规则践行民主法治。积极邀请人大代表、政协委员、群众代表列席旁听政府常务会议，将议决结果向社会发布。对涉及公众重大利益的政策，引入无利害关系、无利益关联的第三方机构拟定草案。对已建成的重点工程、民生项目，开展民意反馈调查。主动落实市人大“专题询问”机制，虚心接受政协民主监督。尊重群众监督政府和权力的热情，建立“社会动员—社会监督—社会管理—社会建设—社会和谐”保障机制。加快完善行政程序，逐一明确各执法环节和步骤及其相应规则，力求行政执法程序规则明确、公开、公正、合理，让生活在宿迁这片土地上的人们处处感受到民主的光辉、法治的力量、正义的温暖。

以制度建设筑牢从政底线。继承发扬艰苦奋斗、勤俭办事的优良传统，做到财政供养人员、“三公经费”只减不增，政府性楼堂馆所一律停建，一般性支出压缩5%以上，做好“三公经费”公开试点。坚持用制度管权、管事、管人，既重视实体性制度建设，又重视程序性制度配套，保证制度行得通、管得住、效果发挥得好。进一步强化反腐倡廉教育和廉政文化建设，严格履行“一岗双责”和廉洁自律各项规定，严肃查处各类违纪违法案件，坚决治理和纠正损害群众利益的不正之风，以干部清正、政府清廉、政治清明赢得广大人民群众的信任、支持和拥护。

各位代表，实干收获希望，拼搏创造未来。我们有信心、有决心、也有能力担负起人民赋予的光荣使命。让我们在市委的坚强领导下，在市人大、市政协和社会各界的监督支持下，同心同德，开拓创新，务实苦干，确保圆满完成全年目标任务，为如期如实建成小康社会、开创宿迁改革发展的新局面而努力奋斗！
